# Significant Differences in Physicochemical Properties of Human Immunoglobulin Kappa and Lambda CDR3 Regions

**DOI:** 10.3389/fimmu.2016.00388

**Published:** 2016-09-27

**Authors:** Catherine L. Townsend, Julie M. J. Laffy, Yu-Chang Bryan Wu, Joselli Silva O’Hare, Victoria Martin, David Kipling, Franca Fraternali, Deborah K. Dunn-Walters

**Affiliations:** ^1^Department of Immunobiology, King’s College London, London, UK; ^2^Randall Division of Cell and Molecular Biophysics, King’s College London, London, UK; ^3^Division of Cancer and Genetics, School of Medicine, Cardiff University, Cardiff, UK; ^4^Faculty of Health and Medical Sciences, University of Surrey, Guildford, UK

**Keywords:** antibody, light chains, kappa, lambda, CDR3, TdT, N nucleotide addition, immunoglobulin

## Abstract

Antibody variable regions are composed of a heavy and a light chain, and in humans, there are two light chain isotypes: kappa and lambda. Despite their importance in receptor editing, the light chain is often overlooked in the antibody literature, with the focus being on the heavy chain complementarity-determining region (CDR)-H3 region. In this paper, we set out to investigate the physicochemical and structural differences between human kappa and lambda light chain CDR regions. We constructed a dataset containing over 29,000 light chain variable region sequences from IgM-transcribing, newly formed B cells isolated from human bone marrow and peripheral blood. We also used a published human naïve dataset to investigate the CDR-H3 properties of heavy chains paired with kappa and lambda light chains and probed the Protein Data Bank to investigate the structural differences between kappa and lambda antibody CDR regions. We found that kappa and lambda light chains have very different CDR physicochemical and structural properties, whereas the heavy chains with which they are paired do not differ significantly. We also observed that the mean CDR3 N nucleotide addition in the kappa, lambda, and heavy chain gene rearrangements are correlated within donors but can differ between donors. This indicates that terminal deoxynucleotidyl transferase may work with differing efficiencies between different people but the same efficiency in the different classes of immunoglobulin chain within one person. We have observed large differences in the physicochemical and structural properties of kappa and lambda light chain CDR regions. This may reflect different roles in the humoral immune response.

## Introduction

Immunoglobulins are a crucial component of the humoral immune system. They are Y-shaped heterodimeric proteins expressed by B cells that are composed of two identical heavy chains and two identical light chains. They can be cell-surface bound as B cell receptors (BCRs) or released into the extracellular environment as antibodies. There is enormous diversity in the immunoglobulin repertoire, which is required to facilitate recognition of a wide variety of different antigen challenges.

Variability in the antigen-binding sites is achieved by V(D)J recombination, combinatorial diversity *via* heavy and light chain pairing, and the post-activation processes of somatic hypermutation and class switching. There are five heavy chain isotypes (IgM, IgD, IgG, IgE, and IgA), which confer different antibody functions, and two light chain isotypes (kappa and lambda). The most diverse immunoglobulin regions are the six hypervariable complementarity-determining region (CDR) loops, which are held in place by the structural beta-sheet framework regions (FRs) (1). The CDR-H3 has a particularly high diversity, arising from a combination of IGHD gene inclusion, extra nucleotide addition by terminal deoxynucleotidyl transferase (TdT), and imprecise joining of the gene segments (2). CDR-H3 is often considered to be the main protein loop involved in antibody specificity (3, 4), and this region can be considered a fingerprint for the B cell and its progeny. The CDR-L3 region is similarly diverse, although without the contribution from a D gene, the degree of variability is less. However, light chains can also be important for the binding specificity of antibodies; light chains are swapped during receptor editing to change the specificity of the antibody (5, 6). Hence, the contribution of light chains to the antigen-binding sites must not be overlooked.

The genes encoding the two light chain isotypes are located on separate chromosomes. Kappa gene segments are encoded on chromosome 2 (7) comprising 52 V genes and 5 J genes (8), whereas lambda gene segments are encoded on chromosome 22 (9) comprising 30 V genes and 7 J genes (10). Kappa locus rearrangement usually precedes the rearrangement of the lambda locus (11), and there are more kappa antibodies in the human peripheral blood, with the kappa/lambda ratio reported to be between approximately 1.5 and 2 (12–14). However, in antigen-selected populations, this ratio can differ significantly depending on the class of antibody heavy chain (15). As an example, antibodies in mucosal secretions (predominantly IgA) have been reported as being mostly lambda (12).

Broad phenotypic differences, such as conformational flexibility (16), half-life (14), and propensity to alter antibody specificity (17), have been noted between antibodies bearing kappa or lambda light chains. There are also reports of altered kappa:lambda ratios being characteristic of certain diseases (18). Notably, it has recently been shown that in chronic HIV patients, HIV Env-specific antibodies have a very strong bias in favor of the lambda light chain (19). Hence, we hypothesize that differential use of kappa and lambda light chains may lead to differing binding specificities, and this may be indicated by inherently different characteristics in the binding regions of the two light chain isotypes.

We have used long read high-throughput sequencing to obtain 29,447 human light chain variable region sequences from antigen-inexperienced cells in order to investigate potential differences between kappa and lambda antigen-binding sites. We compared the kappa and lambda CDR-L3 regions and discovered large, highly significant differences in the physicochemical properties, which were largely encoded in the germline IGLV and IGLJ gene segments. Inclusion of CDR-H3 in the analysis indicates that a correlation between N region additions in all Ig gene rearrangements exists within an individual, but that there is interindividual variation, suggesting variation in TdT activity. Additionally, we have used published human paired heavy and light chain variable sequences (20) to investigate the CDR-H3 properties of heavy chains paired with kappa or lambda light chains and shown that the pairing of heavy and light chain has very little, if any, bias. To assess whether structural differences exist between kappa and lambda light chains, we analyzed antibody structures in the Protein Data Bank (PDB) and observed significant differences in the secondary structure content of the light chain CDR regions.

## Materials and Methods

### Sample Collection

Bone marrow and peripheral blood was collected from 19 healthy donors, aged 24–86, with no known autoimmune disease, undergoing hip replacement surgery at Guy’s Hospital, London, UK (REC# 11/LO/1266).

### Extracting Lymphocytes from Bone Marrow

The bone marrow matrix was removed from the head of the femur by scraping. Cells were washed out of the bone cavity using BM isolation buffer (10 mM EDTA, 2% (v/v) HI-FCS, 1× dPBS, pH 7.4). The sample was transferred to a 50-ml Falcon tube. The volume was made up to 35 ml using BM isolation buffer and mixed by inverting the tube. The mixture was passed through a 100-μm cell strainer (Falcon) into a clean 50-ml Falcon tube. The strained solution was carefully layered onto 15-ml Ficoll (room temperature) and the lymphocytes layered by centrifugation at 400 *g* for 35 min (no brake). The lymphocyte layer was removed into a clean 50-ml Falcon tube and washed once in BM isolation buffer, collecting by centrifugation at 300 *g* for 10 min, and once in 1-ml RPMI suspension buffer (10% HI-FCS, RPMI 1640) in a 1.5-ml microcentrifuge tube, collecting by centrifuging in microcentrifuge at 2 × 1,000 for 5 min. The pellet was re-suspended in 1-ml RPMI suspension buffer, and cells were counted. Cells were frozen overnight in HI-FCS, 10% DMSO at −80°C (Mr. Frosty Freezing Container, Thermo Scientific) before storing in liquid nitrogen until use.

### Extracting Lymphocytes from Peripheral Blood

Ten milliliters of peripheral blood was diluted 1:2 using RPMI suspension buffer. Thirty milliliters of blood suspension was carefully layered onto 15-ml Ficoll in a 50-ml LeucoSep tube (Greiner). After centrifugation at 448 *g*, 20 min (no brake), the lymphocyte layer was removed into a clean 50-ml Falcon tube. Washing in 10-ml RPMI suspension media (centrifugation at 275 *g*, 10 min) was performed twice. Cells were re-suspended in RPMI suspension media, counted, and stored as above.

### B Cell Isolation and Sorting

Bone marrow CD19^+^ B cells were enriched to >98% using anti-human CD19 MicroBeads (Miltenyi Biotec) according to the manufacturer’s protocol. B cell suspensions were blocked (RPMI, 10% normal mouse serum) and subsequently stained using the following antibodies: PE anti-human Ig light chain lambda (MHL-38, Biolegend), APC anti-human Ig light chain kappa (MHK-49, Biolegend), PE/Cy7 anti-human CD38 (HIT2, Biolegend), PerCP/Cy5.5 anti-human IgD (IA6-2, Biolegend), Pacific Blue anti-human IgM (MHM-88, Biolegend), APC/Cy7 anti-human CD10 (HI10a, Biolegend), and CD27-FITC (M-T271, Miltenyi Biotec). The FACS Aria (BD Biosciences) was used to isolate pre-B and immature B cells (IgM^+^IgD^−^CD38^+^CD27^−^CD10^+^) directly into Sort Lysis Reverse Transcription (SLyRT) buffer (21). These two types of B cell were analyzed collectively as “immature B cells” henceforth.

Peripheral blood mononuclear cells (PBMCs) were stained using APC anti-human CD19 (HIB19, BD Bioscience), PerCP/Cy5.5 anti-human IgD (IA6-2, Biolegend), APC/Cy7 anti-human CD10 (HI10a, Biolegend), and CD27-FITC (M-T271, Miltenyi Biotec). The FACS Aria was used to sort naïve (IgD^+^CD27^−^CD10^−^) and transitional (IgD^+^CD27^−^CD10^+^) B cells directly into SlyRT buffer.

FACS plots illustrating the cell sorting are available in Martin et al. (In Review).^1^

### High-Throughput Sequencing and Sequence Analysis

Heavy and light chain variable region cDNA was reverse transcribed from cells in SlyRT buffer, and donor-distinguishing multiplex identifiers (MIDs) were added using semi-nested PCR as described previously (22). Sequencing was carried out on the Roche 454 Titanium platform by LGC Genomics (Germany). Data clean-up was then carried out as previously published (22). Heavy and light chain variable region sequences were not paired. The heavy and light chain sequencing data are available from the Sequence Read Archive (accession number SRP081849).

Sequences then underwent immunoglobulin (Ig) genotyping. Ig gene usage and the CDR amino acid sequences were determined using International Immunogenetics Information System (IMGT) HighV-QUEST (23). The R (24) package Peptides (25) was used to determine the physicochemical properties of CDR peptide sequences. Following IMGT definitions, CDR-H3/-L3 regions were defined as amino acid positions 105–117, CDR-L1: positions 27–38 and CDR-L2: positions 56–65 (26).

Clonotype clustering was carried out on CDR3 regions using the following protocol. Data were split into V family subsets, and the CDR3 nucleotide sequences were used to generate a simple Levenshtein edit distance matrix of all possible pairwise comparisons. The distance matrix was then hierarchically clustered (complete linkage) and the dendrograms cut at 0.05 to release branches that constitute the clones. Scripts which illustrate the clustering used are available at http://www.bcell.org.uk. Once the clusters of related sequences were established, the modal sequence was identified to be used as a representative of this group and was assigned as a reference sequence. Only the reference sequences were used within this analysis to remove any skewing that could have arisen from PCR amplification and ensure that we did not double-count any duplicates arising from multiple mRNA copies.

Data were stored in a Microsoft Excel spreadsheet, and statistical analyses were performed using Microsoft Excel, R 3.2.1 (24), and GraphPad PRISM version 6.07 for Windows, GraphPad Software, La Jolla, CA, USA, www.graphpad.com.

The heavy and light chain master data files can be found at http://www.bcell.org.uk.

### Analysis of Kappa and Lambda Light Chain CDR Physicochemical Properties

Data from all 19 donors were pooled. The dataset was cleaned by removing entries where the CDR-L3 region was longer than 20 amino acids (highly unlikely to be correct CDR-L3 calling by IMGT HighV-QUEST) or were identified by IMGT HighV-QUEST as unproductive. The final dataset contained 20,571 kappa and 8,876 lambda entries (Supplementary Table 1 in the Supplementary Data Sheet). The data were analyzed by cell type and by kappa and lambda isotype. Cumulative frequency histograms were drawn for a variety of CDR-L3 physicochemical properties. The Kolmogorov–Smirnov test (KS test) was used to evaluate differences in the distributions of properties calculated for the kappa and lambda CDR-L3 regions. Multiple *t*-tests, followed by false discovery rate (FDR) correction for multiple testing (*Q* = 1%), were conducted to measure significant differences in amino acid usage.

Principal component analysis (PCA) was performed using the 10 Kidera factors (27) of the CDR-L3 amino acid sequence. PCA was performed using the *prcomp* function in R.

The physicochemical properties of the kappa and lambda CDR-L1 and CDR-L2 regions were also calculated. The CDR-L1 dataset contained 19,413 kappa and 8,419 lambda entries; the CDR-L2 dataset contained 20,004 kappa and 8,631 lambda entries.

The datasets used in these analyses can be found at http://www.bcell.org.uk.

### Heavy Chain Antibody Variable Region Dataset

We only obtained heavy chain data from 12 of the 19 donors. As with the light chains, the dataset was cleaned by removing entries where the CDR-H3 region was longer than 35 amino acids or was identified by IMGT HighV-QUEST as being unproductive. The final dataset contained 29,016 entries (Supplementary Table 1 in the Supplementary Data Sheet).

### Construction of Theoretical “Germline CDR-L3” Regions

IMGT Protein displays (28) were used to obtain the amino acid sequences for the 5′ end (position 105 onward) of each germline *01 allele IGLV and the first 2 3′ amino acids of each germline *01 allele IGLJ. The frequency of recombination of different IGLV and IGLJ genes in the real dataset was determined. The 5′ IGLV amino acid sequences and 3′ IGLJ amino acid sequences were then combined at an equivalent frequency, thus producing a dataset that is reflective of the original dataset, but only containing theoretical “germline CDR-L3” region amino acid sequences (i.e., CDR-L3 regions encoded by the germline IGLV region and germline IGLJ region, with no random nucleotide addition/deletion by TdT). Four hundred twenty entries were removed due to stop codons in the CDR-L3 regions or “not localized” (NL) genes in the dataset. The final dataset contained 20,379 kappa and 8,648 lambda entries. The physicochemical properties of each theoretical “germline CDR-L3” amino acid sequence were calculated. The physicochemical properties of the “germline” and real CDR-L3 regions were compared using the statistical analyses described above.

### Analysis of Paired Heavy–Light Chain Dataset from the Literature

As our dataset contained no heavy–light chain pairing information, we used a recently published dataset (20), which was generated by the Georgiou lab using their innovative technique for paired heavy–light chain sequencing (29, 30). This dataset consisted of paired heavy and light chain information from the naïve repertoires of three donors. We calculated the physicochemical properties of these CDR-H3 regions. We then removed any sequences where the CDR-H3 region was >35 amino acids long or the sequence lacked light chain information or IGHV/IGHJ assignments. The resulting datasets from the three donors contained 13,771 (Donor 1), 26,343 (Donor 2), and 15,193 (Donor 3) sequences. Comparison of the CDR-H3 physicochemical properties of kappa and lambda antibodies was then conducted using the statistical analyses described above.

### Protein Data Bank Structural Analysis

SAbDab (31) was used to build kappa and lambda datasets of human structures from the PDB (32), which had been solved by X-Ray diffraction at a resolution of less than 3Å. Only paired Ig structures (with both heavy and light chains present) were considered. PDB entries were subsequently culled using PISCES (33), according to a maximum mutual sequence identity of 99% to eliminate redundancy.

From this, datasets of 199 kappa and 106 lambda structures were obtained. CDR analyses were carried out on these datasets; however, due to incomplete CDR information in six PDB structures (4LSQ, 4OB5, 4Y5Y, 4HKX, 5D70, and 7FAB), kappa antibody CDR-H1, lambda antibody CDR-H2, and lambda antibody CDR-L2 analyses were instead performed using 197, 105, and 103 entries, respectively.

Secondary structure probabilities for the individual structures were normalized according to the relative CDR length. They were calculated using DSSP (34) for each of the six CDR regions (Chothia definition) (1, 35) in the kappa and lambda datasets. The DSSP algorithm assigns secondary structure to residues according to a hydrogen-bond definition with an energy cut-off of less than −0.5 kcal mol^−1^ (34). The DSSP output was recorded as follows: extended β-strands and β-bridges as “Beta”; α-helices, 3_10_-helices, and π-helices as “Helix”; 3, 4, and 5 turns and non-hydrogen bonded bends as “Turn”; and random coil as “Coil.”

Mean probability values and error bars were calculated using a bootstrapping method [*boot()* function in R] to generate 100 randomly resampled subsets for each of the reference datasets. Error bars were computed as the 95% confidence intervals of the bootstrapped distributions. This permitted estimates of the accuracies of the calculations and avoided any biases resulting from dataset selection.

## Results

### Lambda CDR-L3 Regions Are Significantly Longer and More Hydrophobic than Kappa CDR-L3 Regions

**Figure 1:**
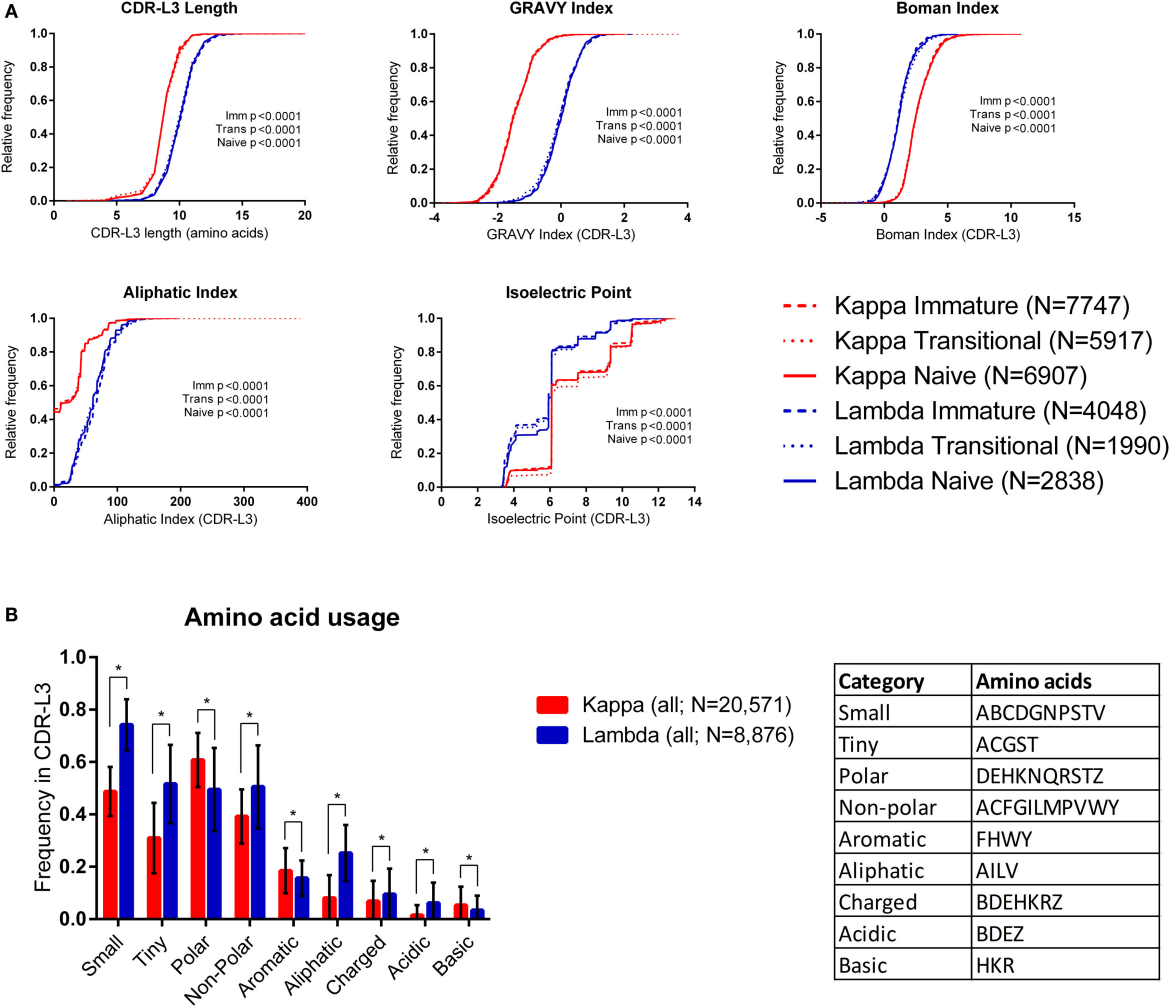
**Physicochemical properties of kappa and lambda CDR-L3 amino acid sequences**. **(A)** Cumulative frequency histograms of a number of physicochemical properties of the kappa (red) and lambda (blue) CDR-L3 regions for each cell type (immature, transitional, and naïve). In every case, the distributions of the kappa and lambda repertoires for each cell type were significantly different (*p* < 0.0001, Kolmogorov–Smirnov test). **(B)** The mean frequency of usage in kappa and lambda CDR-L3 regions of each amino acid class (as described in the table). All cell types were pooled together. Tiny, non-polar, aliphatic, charged, and acidic amino acids are more frequently used in lambda CDR-L3 regions, whereas polar, aromatic, and basic amino acids are more frequently used in kappa CDR-L3 regions. Multiple *t*-tests were performed, followed by false discovery rate (FDR) correction for multiple testing (*Q* = 1%); an asterisk (*) indicates a discovery. Error bars indicate 1 SD.

Figure 1A shows cumulative frequency histograms of the length, hydrophobicity [GRAVY (36) and Boman (37) indices], aliphatic index (38), and isoelectric point (pI) (39) of kappa and lambda CDR-L3 regions of the antibody repertoires from each cell type (immature, transitional, and naïve). In each case, the lambda CDR-L3 regions are significantly longer, more hydrophobic, and have a higher aliphatic index than kappa CDR-L3 regions (*p* < 0.0001; KS test). It was also found, in all three cell types, that lambda light chains have lower pIs on average than kappa light chains (see Supplementary Table 2 in the Supplementary Data Sheet for mean values, SD, and 95% confidence intervals of all CDR-L3 properties measured). Both kappa and lambda repertoires show a “step change” in cumulative frequency at pI 6.08–6.10 due to the very high frequency of occurrence of this characteristic in both repertoires. We also looked at the same physicochemical properties of the CDR-L1 and CDR-L2 amino acid sequences and found significant differences between the kappa and lambda isotypes (Supplementary Figure 1 in the Supplementary Data Sheet). Within the same light chain isotype, there were sometimes small differences in the distributions of CDR properties between the different immature/transition/naïve cell types, although these differences were negligible in comparison to the large differences between kappa and lambda isotypes. We therefore pooled the data from all three cell types (into “kappa” and “lambda”) for subsequent analyses.

We also looked at the frequency of various classes of amino acids in the CDR-L3 regions. Amino acids were classified by their properties as follows (25): tiny (ACGST), small (ABCDGNPSTV), aliphatic (AILV), aromatic (FHWY), non-polar (ACFGILMPVWY), polar (DEHKNQRSTZ), charged (BDEHKRZ), basic (HKR), and acidic (BDEZ). Figure 1B shows that the amino acid composition of the CDR-L3 regions is significantly different between kappa and lambda [multiple *t*-tests and FDR correction (*Q* = 1%)].

### Differences between Kappa and Lambda CDR-L3 Properties Are Encoded in the Germline

**Figure 2:**
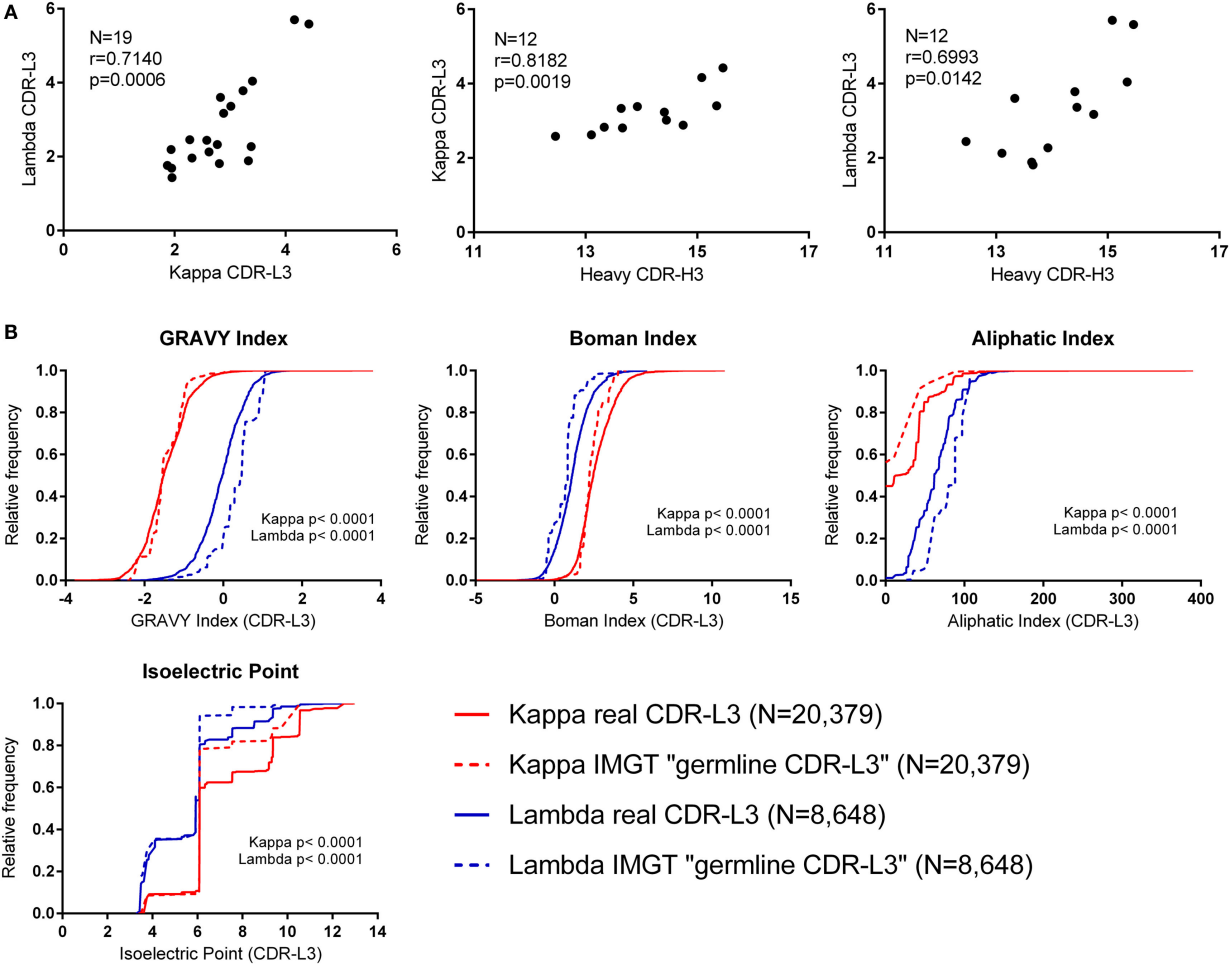
**Investigating the effect of N nucleotide addition and germline sequences**. **(A)** The mean N nucleotide addition per donor (all cell types) (*N* = number of donors). Spearman’s rank correlation coefficient (*r*) and two-tailed *p* values (*p*) show that N nucleotide addition is positively correlated between kappa, lambda, and heavy chain CDR3 regions. **(B)** Physicochemical properties of theoretical “germline CDR-L3” regions (constructed using IMGT sequences) plotted alongside the physicochemical properties of the real CDR-L3 sequences (all cell types). The distributions of the real and IMGT datasets were significantly different for every property measured (*p* < 0.0001, Kolmogorov–Smirnov test). A cumulative frequency histogram for the CDR-L3 length could not be plotted because kappa “germline CDR-L3” regions were all 9 amino acids long; on average, the real kappa CDR-L3 regions were 9.20 amino acids long, and the lambda real and “germline” were 10.48 and 10.83 amino acids long, respectively.

The CDR-L3 region is encoded by germline IGLV/J genes together with a small number of non-germline nucleotides added by the TdT enzyme. We found that the mean number of N nucleotide additions by TdT, as reported by IMGT HighV-QUEST, was slightly higher in lambda than kappa (lambda mean = 3.321, 95% CI [3.204, 3.438] and kappa mean = 3.046, 95% CI [2.990, 3.102]). Interestingly, we observed that the mean number of N additions per donor showed a significant positive correlation between kappa and lambda loci (Figure 2A). Furthermore, the mean number of kappa/lambda light chain N additions per donor was also significantly positively correlated with the mean number of heavy chain N additions in the same donor (Figure 2A). This may indicate that TdT works with differing efficiencies between different people, but the same efficiency in the different classes of immunoglobulin chain. Mean N addition was not significantly correlated with the age of the donor (data not shown). The mean kappa, lambda, and heavy chain N addition differed by as much as 2.5, 4.3, and 3 nucleotides, respectively, between some donors, indicating an approximate difference of 1 non-germline-encoded CDR3 amino acid.

Due to the small number of N additions, and the contribution to the CDR-L3 codons from IGLV and IGLJ genes, the precise effect of N addition/deletion on the amino acid content at the CDR-L3 region cannot be assessed in order to accurately measure any qualitative effect due to TdT/exonuclease activity. Therefore, we concentrated on the amino acid contribution to CDR-L3 from the germline IGLV and IGLJ sequences. To achieve this, we built a dataset that is composed of theoretical “germline CDR-L3” regions, where IMGT germline IGLV and IGLJ amino acid sequences were combined and represented at the same frequency with which they occurred in the real dataset.

Figure 2B shows the theoretical “germline CDR-L3” physicochemical properties plotted against the physicochemical properties of the real dataset. The differences between kappa and lambda are still clearly visible in the theoretical germline dataset, indicating that these physicochemical differences are mostly encoded in the germline. We can see differences between the real and theoretical datasets, implying that nucleotide addition/deletion does have a significant effect on the physicochemical properties of the CDR-L3 region, although palindromic (P) nucleotide addition would also contribute to this difference. The effect of the addition/deletion of nucleotides is not consistent between kappa and lambda genes (i.e., the difference between kappa and lambda in the germline dataset is not consistently bigger or smaller than in the real dataset).

### Kappa and Lambda Isotypes Are Separated by PCA Using CDR-L3 Region Kidera Factors

**Figure 3:**
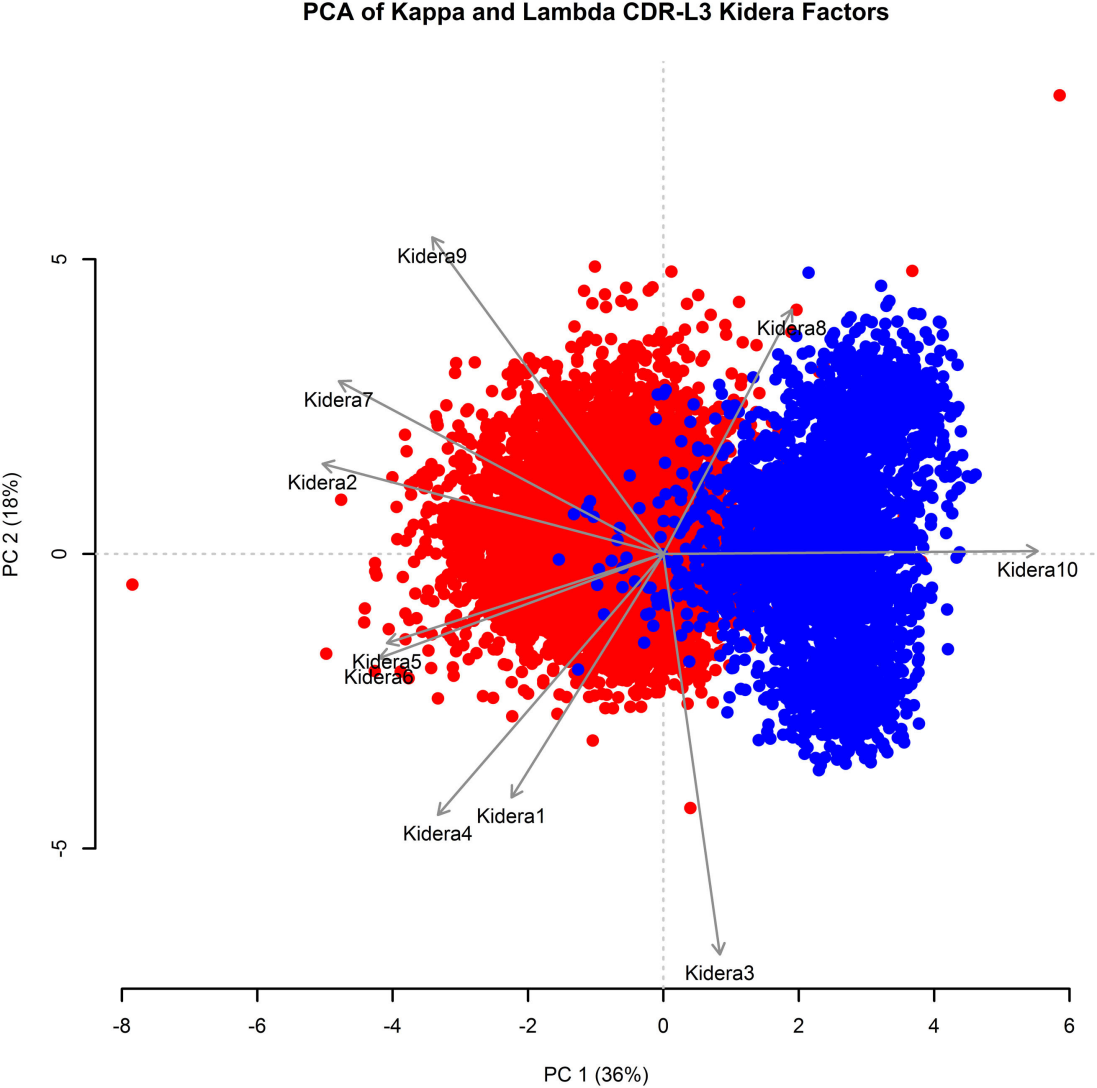
**Principal component analysis (PCA) using Kidera factors of the light chain CDR-L3 amino acid sequences**. PCA was performed using the Kidera factor vectors 1–10 of each kappa (red, *N* = 20,571) and lambda (blue, *N* = 8,876) CDR-L3 amino acid sequence. PC1 shows separation of the kappa and lambda isotypes. The Kidera factors that were most correlated with PC1 are Kidera 10, Kidera 2, and Kidera 7.

Kidera factors are a set of 10 factors, which describe orthogonal physicochemical protein properties that are related to protein structure (27, 40). Figure 3 shows a PCA conducted using the 10 Kidera factors for the CDR-L3 region of each of the 20,571 kappa sequences and 8,876 lambda sequences. Principal component 1 (PC1) splits the kappa and lambda isotypes into two clusters. The Kidera factors that were best correlated with PC1 were Factor 10 (surrounding hydrophobicity), Factor 2 (side-chain size), and Factor 7 (flat extended preference) (41). The cumulative frequency distributions of kappa and lambda CDR-L3 Kidera factors can be found in Supplementary Figure 2 in the Supplementary Data Sheet.

Some separation of the kappa and lambda isotypes can be seen when applying PCA to CDR-L1 and CDR-L2; however, the separation is not as clear as it is in the CDR-L3 region (data not shown).

### The Distributions of CDR-H3 Properties in Kappa and Lambda Antibodies Are Not Consistently Different

**Figure 4:**
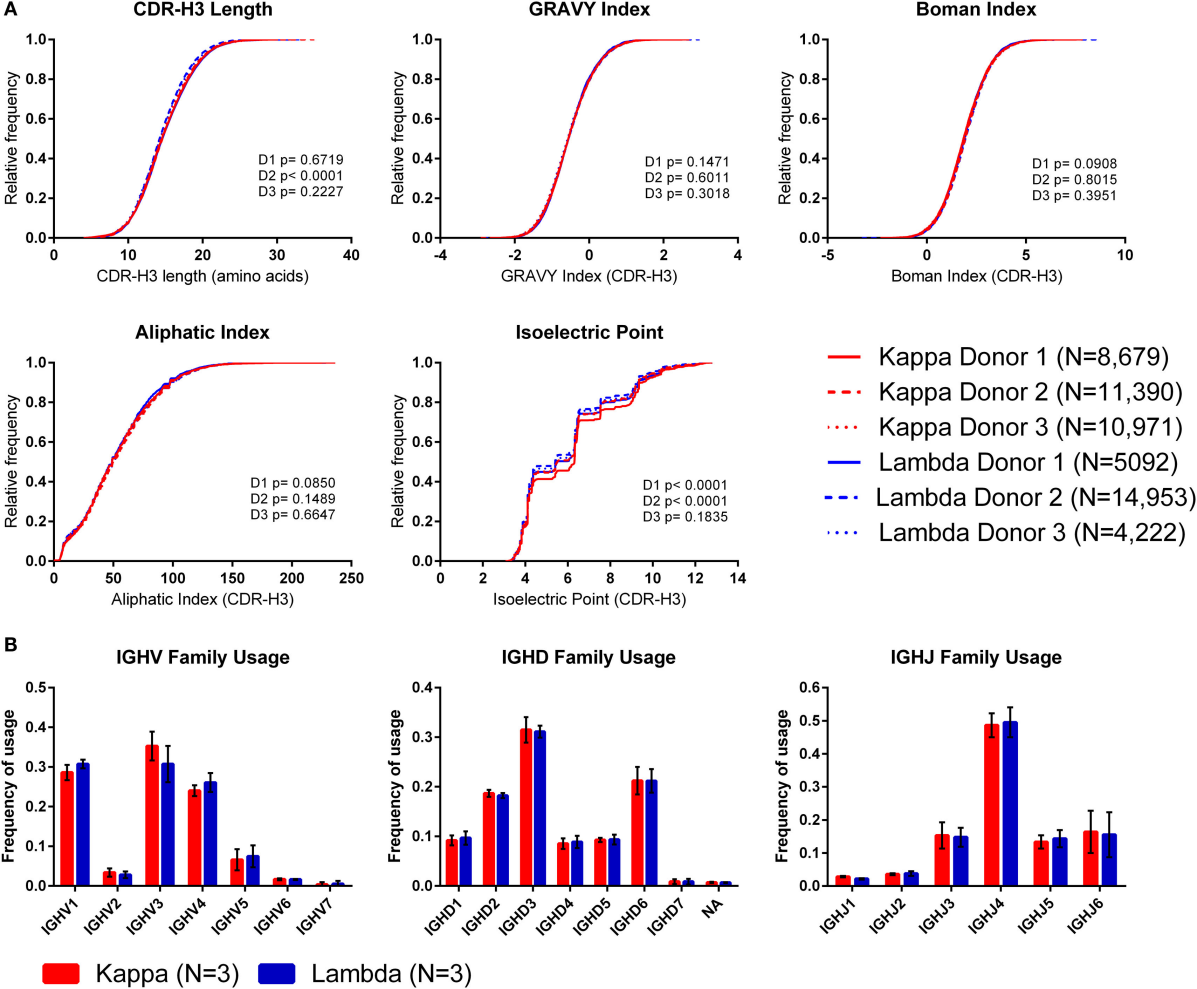
**The CDR-H3 physicochemical properties and IGHV-D-J usage of heavy chains paired with kappa or lambda light chains**. **(A)** Cumulative frequency histograms of CDR-H3 physicochemical properties of kappa and lambda antibodies. Each donor is plotted separately. Statistical significance for each donor was calculated using the Kolmogorov–Smirnov test. D1, D2, and D3 refer to Donor 1, Donor 2, and Donor 3, respectively. **(B)** Mean frequency of IGHV-D-J family usage across the three donors. Error bars show ±1 SD.

To investigate whether the use of kappa or lambda is associated with particular heavy chain properties, we measured a variety of physicochemical properties of CDR-H3 amino acid sequences obtained from the paired heavy–light chain variable region sequences of naïve B cells from three donors (20) and compared the values from heavy chains paired with kappa light chains to heavy chains paired with lambda light chains (see Supplementary Figure 3 in the Supplementary Data Sheet for cumulative frequency plots, which confirm that the light chain isotypes of this published dataset separate in the same way as those from our dataset, as shown in Figure 1A). To account for donor variability, we plotted the kappa and lambda antibody CDR-H3 repertoires of each donor separately (Figure 4A). We then compared the kappa and lambda CDR-H3 property distributions for each donor individually using the KS test. We found that for many of the properties (e.g., GRAVY index, Boman index, and aliphatic index), there was no significant difference between the repertoires for any of the donors. However, we did find that for Donor 2, the lambda repertoire CDR-H3 regions were significantly shorter than the kappa repertoire, and for Donors 1 and 2, we found that the lambda repertoire had a significantly lower CDR-H3 pIs than the kappa repertoire. We also looked at IGHV-D-J family usage in the kappa and lambda repertoires of the three donors (Figure 4B) and found no significant difference in frequency [multiple *t*-tests and FDR correction (*Q* = 1%)]. The small differences in CDR-H3 physicochemical properties appear to be donor-specific with no overarching effects. This leads us to believe that despite the large differences between kappa and lambda CDR-L3 physicochemical properties, heavy–light chain pairing is virtually random, although there may be some very subtle biases which are specific to individuals.

### Kappa and Lambda Antibodies Are Structurally Different

Figure 3 showed that Kidera factors, which are related to protein structure, separate the kappa and lambda isotypes well. This raised the question: how do the kappa and lambda isotypes influence the light chain structure?

**Figure 5:**
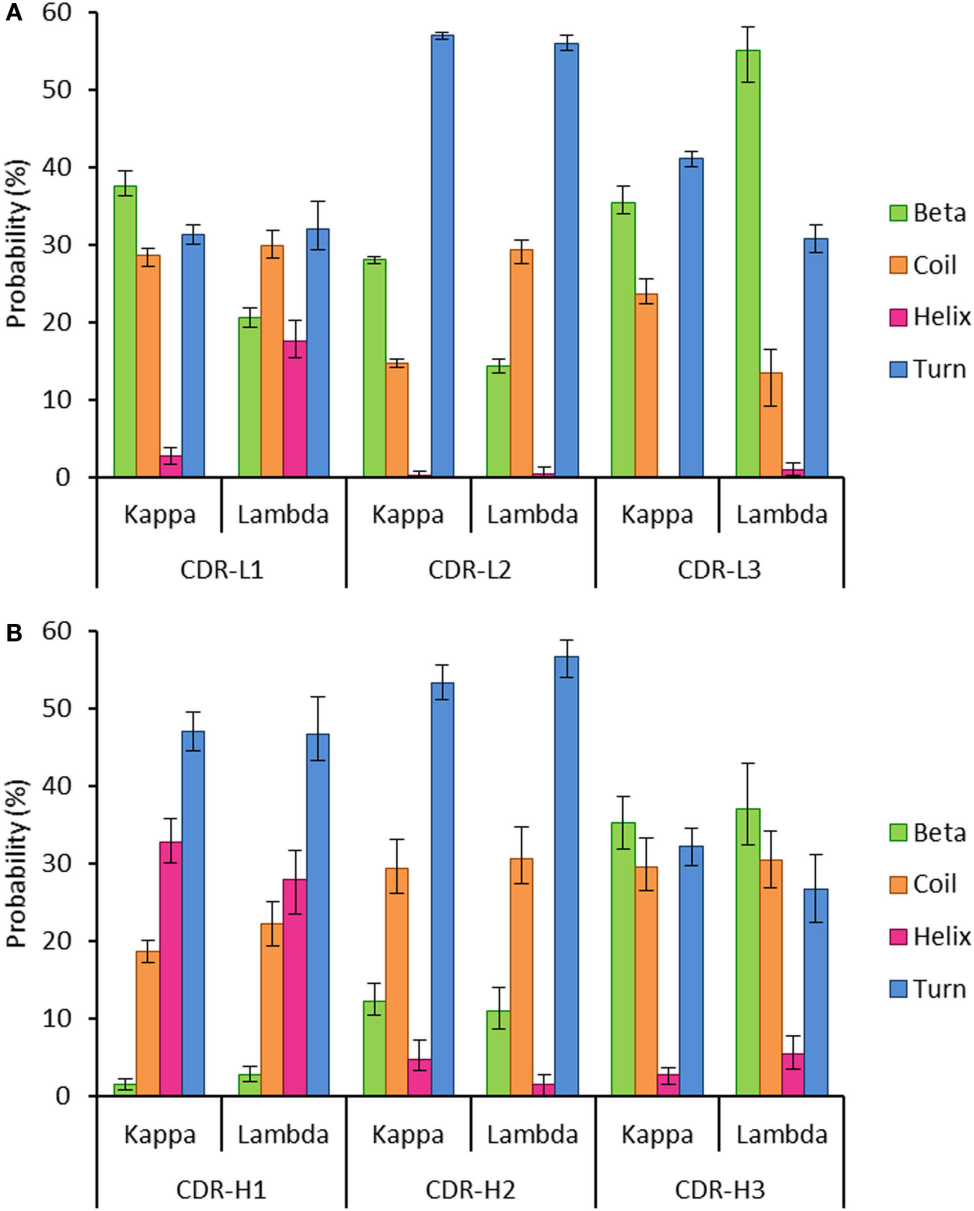
**Probability of encountering various secondary structures in antibody heavy and light chain CDR regions**. The graphs show the mean proportion of residues in the CDR loops, which are conforming to each of the following groups of secondary structures: beta, coil, helix, or turn. The error bars represent the confidence intervals at the 95% level, estimated using 100 runs of bootstrap resampling. **(A)** All the three light chain CDR regions show differences between the kappa and lambda isotypes in the proportion of the CDR regions that are composed of four secondary structures (beta, coil, helix, and turn). **(B)** The only significant difference between kappa and lambda in the composition of heavy chain CDR regions was in the frequency of helix structure in the CDR-H2 region. Differences were considered significant if the 95% CIs did not overlap.

To investigate this, we extracted two datasets of antibody structures from the PDB split according to light chain isotype (kappa or lambda) and calculated the secondary structure occupancies of the light chain CDR regions for each dataset (Figure 5). We found that the beta structure content of kappa light chains was significantly higher than lambda light chains in the CDR-L1 and -L2 regions but significantly lower in CDR-L3 regions (Figure 5A). Differences in beta structure content were compensated for by changes in coil structure content in the CDR-L2 and -L3 regions and, surprisingly, by changes in the helix structure content of the CDR-L1. In this context, significant differences in turn structure (which included 3, 4, and 5 turns and non-hydrogen-bonded bends) were not meaningful as these conformational states serve to link more regular secondary structure types, beta sheets and helices alike. We postulated that secondary structure probabilities could be influenced by CDR length with, for example, shorter polypeptide regions favoring more ordered secondary structures. However, secondary structure probabilities did not appear to correlate with CDR length (data not shown). The secondary structure propensities of heavy chain CDR regions were also compared; however, the only significant difference observed between heavy chain partners of the kappa and lambda light chain isotypes was the proportion of helix in CDR-H2 (Figure 5B). Otherwise, in agreement with the result in Figure 4, all other properties were not significantly different.

## Discussion

In this paper, we have shown that kappa and lambda CDR-L3 regions differ significantly in their physicochemical properties, indicating that kappa and lambda light chains may have differing roles in antibody binding.

In accordance with previous publications, we found that lambda CDR-L3 regions are, on average, significantly longer and more hydrophobic than kappa (20), and, in addition, we found that lambda CDR-L3 regions have a higher aliphatic index than kappa CDR-L3 regions. In the case of heavy chains, long hydrophobic CDR-H3 regions are selected against as B cells mature and pass through tolerance checkpoints (42–44) (and data not shown), and it is thought that these properties in heavy chains are associated with autoreactivity (45). However, despite their longer, hydrophobic qualities, lambda light chains have been reported to be better at removing a polyspecific antibody phenotype than kappa *in vitro* (17). Since, *in vivo*, the lambda locus is rearranged after the kappa locus (13), lambda light chains are perhaps likely to have a role in “rescuing” antibodies that were autoreactive when the same heavy chain was paired with a kappa light chain. The apparent contradiction between proposed roles of longer hydrophobic CDR3 regions in heavy vs. light chains may indicate that CDR-L3 and CDR-H3 regions have different roles to play in the structure of the antigen-binding site.

Both kappa and lambda isotypes had a very high frequency of CDR-L3 regions with pI of between 6.08 and 6.10. A similar pattern is also seen in the heavy chain repertoire in our data (not shown) and Figure 4A. This suggests that a slightly acidic CDR-L3 pI (net negative charge at neutral pH) is highly advantageous in the antibody repertoire and that a CDR-L3 pI of approximately 7.00 (net neutral charge at neutral pH) is less so, as very few sequences of either isotype have a neutral CDR-L3 pI. It has been suggested that a more basic CDR-H3 pI may be associated with polyspecificity (46). Since lambda CDR-L3 regions had a lower mean pI and higher acidic amino acid usage than kappa (Figure 1), the aforementioned ability of lambda light chains to rescue polyspecific antibodies may be a result of this charge-related phenotype. Light chains with a low CDR pI, or high aspartic acid usage, have been shown to be good at rescuing DNA-reactive heavy chains (47, 48). This may be because DNA has a high negative charge; so, reducing the positive charge of an antibody by changing the light chain for one with more acidic character could help abolish inappropriate ionic interactions.

Another possibility to account for a functional difference in kappa and lambda light chains is not that they evolved to bind to different types of antigen, but that they have evolved to respond in different ways to antigen. Codon usage in kappa and lambda light chains leads to quite different affinity maturation patterns as a result of somatic hypermutation; codons encoding the kappa CDRs are prone to more non-conservative mutations than lambda; however, this is also more likely to result in stop codons (49). So, the consequences of somatic hypermutation for kappa and lambda may be quite different, and this could provide a useful extra level of diversity in response to different antigenic challenges.

The kappa/lambda CDR-L3 physicochemical differences that we observed are mostly encoded in the germline, and since it is thought that light chains diverged into isotypes more than 450 million years ago, these differences are the result of millennia of evolution (50). Other species vary with respect to antibody light chains. Bony fish and amphibians are endowed with three distinct light chain isotypes (kappa, lambda, and sigma), whereas mammals and reptiles only possess two (kappa and lambda). The kappa light chain has been lost in birds, leaving only the lambda light chain (51, 52), and camelids have been found to have antibody classes which lack light chains completely (53). This disparity in light chains between species indicates that having two light chain isotypes is not essential for a fully functioning antibody repertoire. However, the fact that camelid heavy chain antibodies require alternative mechanisms of antibody diversification in the absence of light chains indicates that the additional variability that light chains enable is advantageous (54). Recent work in the study of allergy has also highlighted a potential role for free light chains in the antigen-specific activation of mast cells (55).

The N nucleotide addition in light chain CDR-L3 regions is quite limited, but we did see that lambda light chains have a slightly higher mean N addition than kappa. What was most striking was a significant positive correlation of mean N addition between the kappa, lambda, and heavy chain CDR3 regions within individuals, even though there was a mean difference of approximately three non-germline-encoded N nucleotides in the CDR3 regions between some people. We hypothesize that this may be due to individual variation in the efficiency of the N nucleotide addition process. It would be interesting to investigate whether variation in N nucleotide addition affects the humoral immune response.

Heavy–light chain pairing is a subject of great interest, with some groups reporting small biases in pairing (56), whereas others report that the heavy–light chain pairing is not significantly different to that which would be expected if it were random (20, 57). These studies have looked at heavy and light chain V(D)J pairings, whereas we looked at CDR3 physicochemical properties. Overall, we only found a few, donor-specific, significant differences in the distributions of CDR-H3 properties of heavy chains paired with kappa and lambda light chains. Our study supports a hypothesis for virtually random heavy–light chain pairing, although certain small biases can occasionally be seen within individuals.

Structural differences in the CDR loops of kappa and lambda antibodies have been noted previously, notably that the majority (approximately 80%) of kappa CDR-L3 loops have the same canonical structure, whereas lambda CDR-L3 loops can adopt a plethora of canonical structures (58). When analyzing the mean proportions of light chain CDR regions adopting specific secondary structures, we found significant differences between kappa and lambda (Figure 5A). In particular, we found that the likelihood of beta structure content was significantly higher in kappa CDR-L1 and -L2 regions but significantly lower in CDR-L3 regions when compared to lambda light chains. Moreover, we found that differences in the frequency of beta propensity in CDR-L1 and -L2 tended to be compensated for by higher frequencies of helix and coil propensities, respectively, in the lambda isotype. In the CDR-L3 region, there were significant differences between kappa and lambda propensities for beta, coil, and turn structures.

To date, much attention has been paid to the role of the heavy chain CDR-H3 region in antibody binding, and the contribution of the light chain to antibody-binding specificity has sometimes been overlooked. Our findings show that there are significant differences in kappa and lambda light chains and lend further support to a growing body of evidence that they may have different roles in the adaptive immune response.

## Ethics Statement

The study was approved by the Research Ethics Committee London – Camberwell St Giles REC# 11/LO/1266.

## Author Contributions

CT performed the work and analyzed the data. DD-W directed the work. JSO and Y-CW collected samples and performed experiments. VM collected samples. DK, JL, and FF analyzed the data. CT, DD-W, VM, and JL wrote the paper. DD-W takes responsibility for the work.

## Conflict of Interest Statement

A portion of CLT’s PhD stipend is paid by MedImmune, a subsidiary of AstraZeneca.

## Footnotes

^1^Martin MG, Wu YCB, Townsend CL, Lu GH, Silva O’hare J, Mozeika A, et al. Transitional B cells in early human B cell development – time to revisit the paradigm? *Front Immunol* (In Review).
