# Supplementary material for: Significant Differences in Physicochemical Properties of Human Immunoglobulin Kappa and Lambda CDR3 Regions
Source: Front Immunol. 2016 Sep 27;7:388. doi: 10.3389/fimmu.2016.00388 (PMC5037968; doi:10.3389/fimmu.2016.00388)
Supplement: Supplementary file 1 [file Data_Sheet_1.pdf]

Supplementary Material

Significant differences in physicochemical properties of human immunoglobulin kappa and lambda CDR3 regions

Catherine L. Townsend[1], Julie M.J. Laffy[2], Yu-Chang Wu[1], Joselli Silva O'Hare[1], Victoria Martin[1], David Kipling[3], Franca Fraternali[2], Deborah K. Dunn-Walters[1,4*]

***Correspondence:** Deborah Dunn-Walters: d.dunn-walters@surrey.ac.uk

1 Supplementary Tables

		Number of sequences		
**Donor ID**	**Donor Age**	**Kappa**	**Lambda**	**Heavy**
122	24	1464	291	1630
159	28	1482	383	2610
138	41	909	121	2628
107	43	1207	562	0
120	44	841	526	0
118	49	695	323	2313
146	50	1039	364	3905
128	52	606	383	0
103	52	688	316	1646
126	53	1557	735	0
141	65	2039	1048	2570
105	67	661	395	0
119	68	2178	1118	4172
160	70	899	181	2643
111	71	773	503	1380
149	72	729	489	1931
132	76	923	399	0
162	78	771	190	0
140	86	1110	549	1588
	**Total**	**20571**	**8876**	**29016**

Supplementary Table 1: Donor age and count of the number of sequences used in the analyses. The age of all 19 donors and the number of sequences contributed per donor for the kappa, lambda and heavy chain datasets used in the analysis.

	Kappa CDR-L3 (N=20,571)				Lambda CDR-L3 (N=8,876)			
	Mean	SD	L 95% CI	U 95% CI	Mean	SD	L 95% CI	U 95% CI
**CDR-L3 length (AA)**	9.199	1.193	9.183	9.215	10.533	1.277	10.507	10.560
**GRAVY Index**	-1.459	0.584	-1.467	-1.451	-0.049	0.574	-0.061	-0.037
**Boman Index**	2.654	1.134	2.638	2.669	1.161	1.105	1.138	1.184
**Aliphatic Index**	26.778	30.212	26.365	27.191	63.536	27.382	62.966	64.106
**Isoelectric Point**	7.244	2.166	7.214	7.273	5.673	1.786	5.636	5.710
**Frequency Small AA**	0.487	0.094	0.486	0.489	0.742	0.097	0.740	0.744
**Frequency Tiny AA**	0.310	0.134	0.308	0.312	0.516	0.149	0.513	0.519
**Frequency Polar AA**	0.608	0.103	0.606	0.609	0.495	0.159	0.492	0.498
**Frequency Non-Polar AA**	0.392	0.103	0.391	0.394	0.505	0.159	0.502	0.508
**Frequency Aromatic AA**	0.185	0.086	0.184	0.186	0.156	0.068	0.154	0.157
**Frequency Aliphatic AA**	0.080	0.087	0.079	0.081	0.252	0.108	0.250	0.254
**Frequency Charged AA**	0.068	0.078	0.067	0.069	0.095	0.098	0.093	0.097
**Frequency Acidic AA**	0.015	0.039	0.014	0.015	0.061	0.079	0.059	0.063
**Frequency Basic AA**	0.053	0.071	0.052	0.054	0.034	0.056	0.033	0.035
**Kidera 1**	0.448	0.253	0.444	0.451	0.285	0.262	0.279	0.290
**Kidera 2**	0.018	0.260	0.014	0.021	-0.466	0.212	-0.470	-0.461
**Kidera 3**	0.158	0.216	0.155	0.161	0.219	0.346	0.211	0.226
**Kidera 4**	0.218	0.202	0.216	0.221	-0.017	0.215	-0.021	-0.012
**Kidera 5**	0.165	0.173	0.162	0.167	-0.178	0.198	-0.182	-0.174
**Kidera 6**	-0.087	0.194	-0.089	-0.084	-0.293	0.184	-0.297	-0.289
**Kidera 7**	0.019	0.214	0.016	0.022	-0.621	0.310	-0.628	-0.615
**Kidera 8**	-0.424	0.272	-0.428	-0.421	-0.196	0.352	-0.203	-0.189
**Kidera 9**	-0.142	0.261	-0.145	-0.138	-0.413	0.202	-0.417	-0.409
**Kidera 10**	-0.505	0.228	-0.508	-0.501	0.050	0.194	0.046	0.055

Supplementary Table 2: Statistics for the measured CDR-L3 properties. The mean, standard deviation (SD), lower 95% confidence interval (L 95% CI) and upper 95% confidence interval (U 95% CI) of the kappa and lambda repertoires (all cell types). AA = amino acids.

3 Supplementary Figures

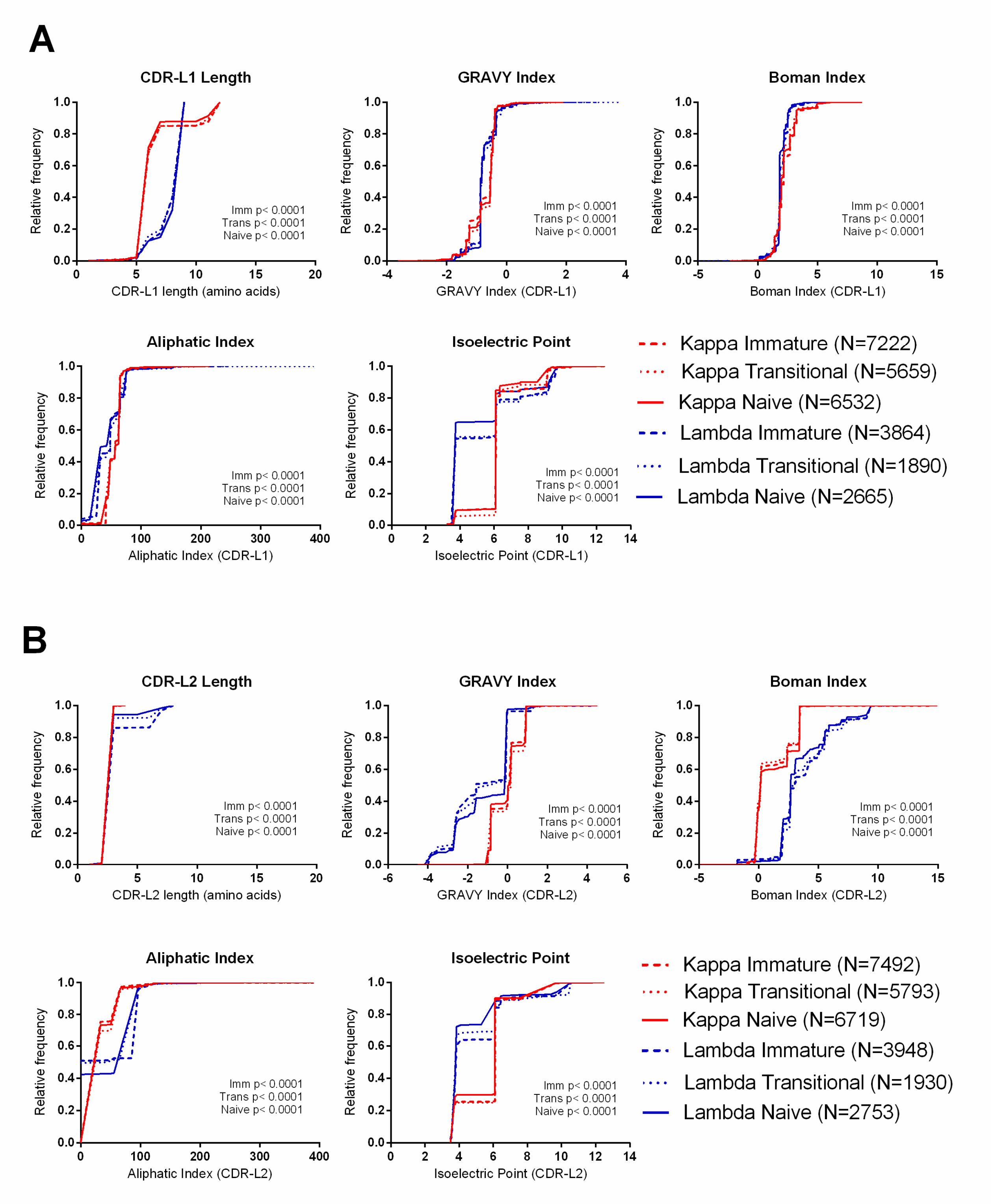

Supplementary Figure 1: Physicochemical properties of kappa and lambda CDR-L1 and CDR-L2 regions. A) Physicochemical properties of CDR-L1 regions. **B)** Physicochemical properties of CDR-L2 regions. All CDR-L1 and CDR-L2 physicochemical properties are significantly different ($p<0.0001$; KS test) between kappa and lambda isotypes. CDR-L1 and CDR-L2 are encoded within the IGLV gene so there is a lot less variation in these CDR regions compared to the CDR-L3; there are therefore many duplicate values, resulting in irregular distributions.

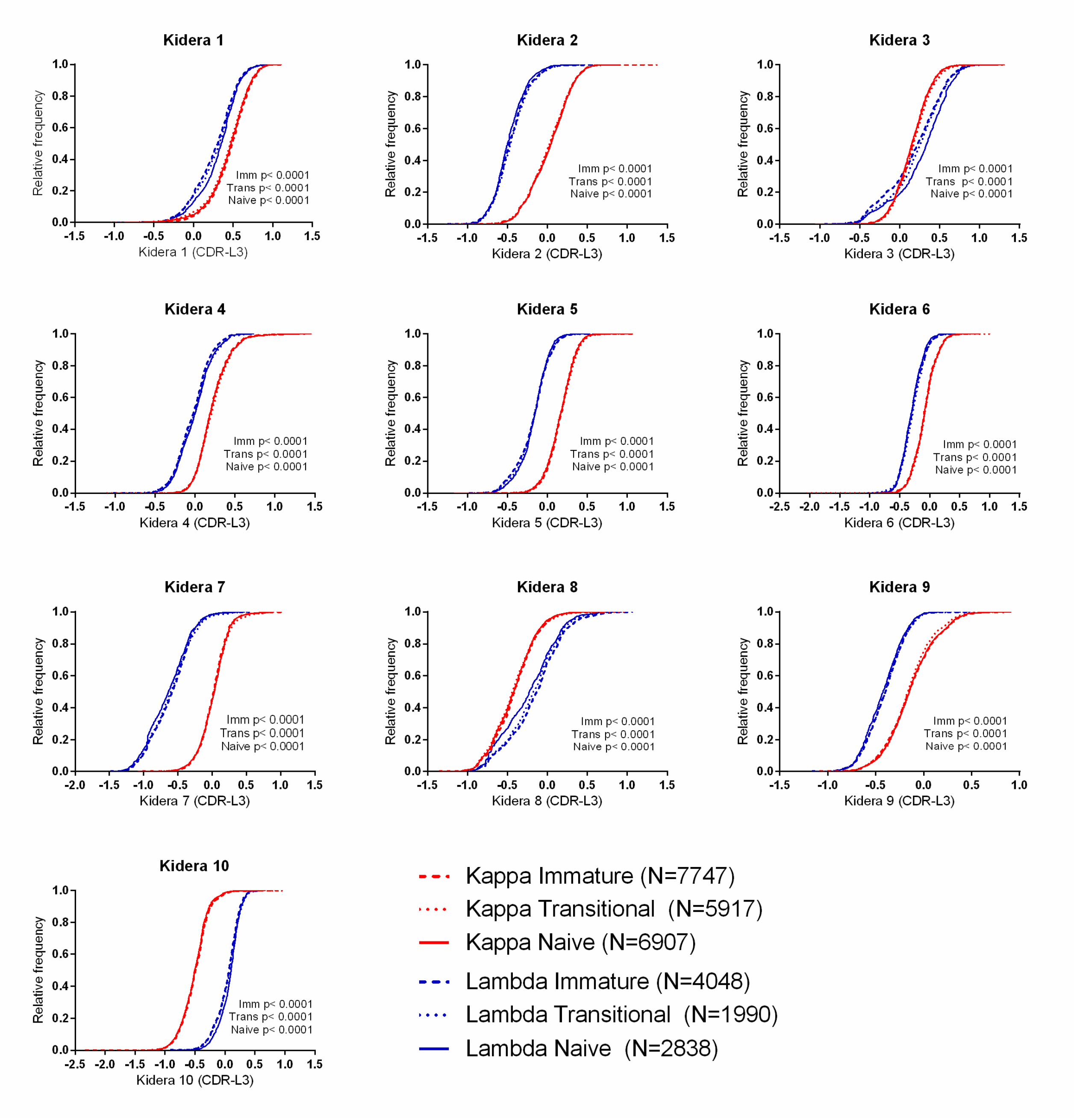

Supplementary Figure 2: Kappa and lambda CDR-L3 Kidera factors. Cumulative frequency histograms of the ten Kidera factors of kappa (red) and lambda (blue) CDR-L3 regions for each cell type (immature, transitional and naïve). In every case, the distributions of the kappa and lambda repertoires for each cell type were significantly different (p<0.0001, Kolmogorov-Smirnov test).

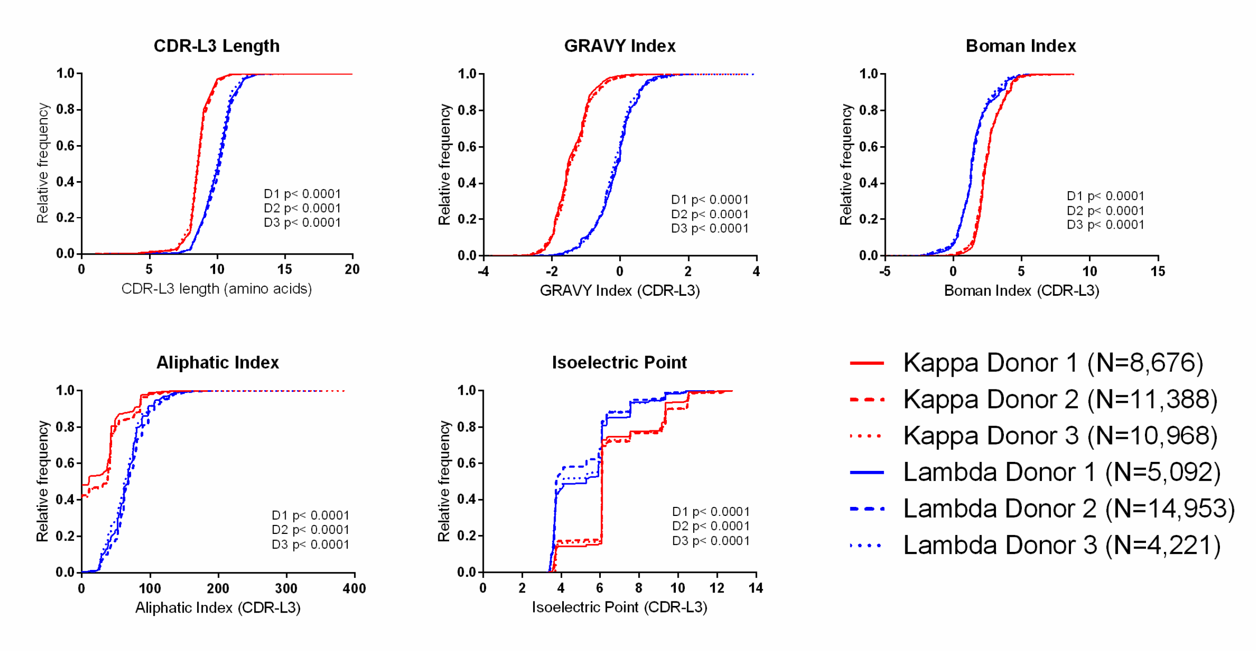

Supplementary Figure 3: Physicochemical properties of CDR-L3 regions from light chains from the literature. Kappa and lambda CDR-L3 regions separate in the same way as the kappa and lambda CDR-L3 regions from our dataset shown in Figure 1A.
